# Surgical management of displaced femoral neck fractures in patients with dementia: a comparison in mortality between hemiarthroplasty and pins/screws

**DOI:** 10.1007/s00068-021-01640-0

**Published:** 2021-04-11

**Authors:** Ioannis Ioannidis, Ahmad Mohammad Ismail, Maximilian Peter Forssten, Rebecka Ahl, Yang Cao, Tomas Borg, Shahin Mohseni

**Affiliations:** 1 Department of Orthopedic Surgery, Orebro University Hospital, 701 85 Orebro, Sweden; 2 Division of Trauma and Emergency Surgery, Department of Surgery, Orebro University Hospital, Orebro, 701 85 Sweden; 3 School of Medical Sciences, Orebro University, 702 81 Orebro, Sweden; 4 Division of Trauma and Emergency surgery, Department of Surgery, Karolinska University Hospital, 171 76 Stockholm, Sweden; 5 Clinical Epidemiology and Biostatistics, School of Medical Sciences, Orebro University, 70182 Orebro, Sweden

**Keywords:** Femoral neck fracture, Hip fracture, Dementia, Hemiarthroplasty, Mortality

## Abstract

**Introduction:**

Dementia is common in patients with hip fractures and is strongly associated with increased postoperative mortality. The choice of surgical intervention for displaced femoral neck fractures (dFNF) in patients with dementia has been a matter of debate. This study aims to investigate how short- and long-term mortality differs between those who have been operated with hemiarthroplasty or pins/screws.

**Methods:**

All patients with dementia and dFNF, i.e., Garden III and IV, who underwent primary emergency hip fracture surgery, with either hemiarthroplasty or pins/screws, in Sweden between Jan 1, 2008 and Dec 31, 2017 were eligible for inclusion in the current study. Patients were divided into two groups based on the surgical intervention: hemiarthroplasty and pins/screws. The primary outcome of interest was 30-day postoperative mortality, and the secondary outcome was 1-year postoperative mortality. Poisson and Cox regression analyses were performed both before and after propensity score matching.

**Results:**

A total of 9394 cases met the inclusion criteria; 84% received hemiarthroplasty and 16% received pins/screws. In the unmatched analysis, the adjusted incidence rate ratio (IRR) for 30-day postoperative mortality was not affected by the chosen surgical method (adj. IRR 0.96, CI 95% 0.83–1.12, *p* = 0.629). After propensity score matching, similar results were observed with no difference in 30-day postoperative mortality (adj. IRR 0.89, CI 95% 0.74–1.09, *p* = 0.286). There was a statistically significant decrease in the risk of 1-year postoperative mortality in the hemiarthroplasty group compared to the pins/screws group, both before and after propensity score matching.

**Conclusion:**

This study could not demonstrate any difference in 30-day mortality in patients with dementia and dFNFs when comparing hemiarthroplasty with pins/screws. Patients that received hemiarthroplasties did, however, have a lower risk of 1-year postoperative mortality.

## Introduction

The lifetime risk of suffering a hip fracture after the age of 50 is approximately 23% for women and 11% for men [1]. Sweden has an annual incidence of over 17,000 hip fractures resulting in an estimated direct cost of 1.5 billion SEK (170 million USD/ 145 million EUR) for the healthcare system [2]. These numbers are expected to continue to grow as the population ages [3]. More than 50% of all hip fractures affect the neck of the femur, of which approximately 70–75% are displaced femoral neck fractures (dFNF) [4]. Furthermore, approximately 23% of all hip fracture patients in Sweden suffer from dementia [5]. By the year 2050, the prevalence of dementia in Sweden, which has been strongly linked to worse outcomes after hip fracture surgery, is expected to double according to The Swedish National Board of Health and Welfare [6, 7].

The choice of surgical intervention for dFNF in patients with dementia, who are often both frail and have several comorbidities, is a matter of much debate. Some argue that hemiarthroplasty results in better postoperative quality of life, whereas others argue that the anesthesia, along with the “extended” surgical trauma associated with hemiarthroplasty in comparison to internal fixation with pins/screws, may result in worse short-term outcomes [8]. Consequently, this study aims to compare the difference in short- and long-term mortality in patients with dementia who undergo surgery with hemiarthroplasty or pins/screws for dFNF.

## Materials and methods

The principles of the Declaration of Helsinki and the STROBE guidelines were adhered to while conducting this study. Ethical approval was obtained from the National Swedish Review Authority (reference number 2020–04,161). The study cohort was obtained from the Rikshoft register, the Swedish National Quality Registry for Hip Fracture Patients and Treatment, which is composed of prospectively collected data [9]. All adults with the diagnosis of dementia who underwent emergency hip fracture surgery for dFNF, i.e. Garden III and IV, in Sweden between Jan 1, 2008 and Dec 31, 2017 were considered for inclusion in the study. Cases where the hip fractures were pathological or conservatively managed were not included in the data retrieval. In addition, patients with missing data for arrival time and time of surgery were excluded from analysis. The data from Rikshoft were used to determine the date of hospital admission, age, sex, fracture type, American Society of Anesthesiologist (ASA) classification, surgical method, date of surgery, and hospital discharge date. These data were then cross-referenced with the Swedish National Board of Health and Welfare registers using the patient’s social security numbers, to collect information on time of death and comorbidity data. The comorbidity data were used to calculate the Charlson Comorbidity Index (CCI) for each patient [10].

### Statistical analysis

The cases were divided into two groups: hemiarthroplasty and pins/screws. Demographics, clinical characteristics, and outcomes were compared between the two groups. Categorical variables were reported with percentages while continuous variables were reported as a mean ± standard deviation (SD) or median and interquartile range (IQR). Pearson’s chi-squared test and Fisher’s exact test were used to determine the statistical significance of differences between unmatched categorical variables; McNemar’s test with Bonferroni correction was used for matched variables. For unmatched continuous variables, the Student’s *t* test was used for normally distributed data, otherwise Mann–Whitney *U* test was applied; if the variable was matched, the paired version of these tests was used instead. The primary outcome of interest was 30-day postoperative mortality. The secondary outcome of interest was 1-year postoperative mortality.

A Poisson regression model was employed to investigate the association between the surgical method used and 30-day postoperative mortality while a Cox proportional hazards model was used to investigate the association with mortality 1-year postoperatively. All analyses were performed while adjusting for age, sex, time to surgery, ASA class, and comorbidities. Results for 30-day mortality are reported as incidence rate ratios (IRR) with 95% confidence intervals (CI) while results for 1-year mortality are reported as hazard ratios (HR) with 95% CIs.

The cohorts were also matched at a 1:1 ratio using propensity score matching. Nearest neighbor matching with a caliper of 0.2 was selected as the matching algorithm. Variables included in the propensity score matching were sex, age, ASA classification, myocardial infarction, cerebrovascular disease, peripheral vascular disease, chronic obstructive pulmonary disease, congestive heart failure, connective tissue disease, diabetes, liver disease, local tumor, metastatic cancer, and chronic kidney disease. A conditional Poisson regression model and a Cox regression model with shared frailty were used for the matched cohorts when analyzing 30-day and 1-year postoperative mortality, respectively.

Statistical significance was defined as a two-sided *p* value less than 0.05. Analyses were performed using the statistical programming language R (R Foundation for Statistical Computing, Vienna, Austria) and Stata 16.1 (StataCorp, College Station, Texas, USA) [11].

## Results

During the study period, a total of 27,887 cases with dementia and traumatic hip fractures were registered in Rikshoft. Of these, 9394 (34%) were operated for a dFNF, using hemiarthroplasty or pins/screws, and were included in the study. The mean age of the cohort was 85 years and there was no clinically significant difference in age when comparing patients who received pins/screws to those who received hemiarthroplasty (84 ± 9 years vs. 85 ± 6 years, *p* < 0.001). The pins/screws group was operated on within 24 h more often than the hemiarthroplasty group (69.8 vs 63.7%, *p* < 0.001). The patients who received pins/screws were less fit for surgery (ASA ≥ 4 18.4 vs 8.1%, *p* < 0.001) and had more comorbidities (CCI ≥ 7 30.0 vs 28.5%, *p* < 0.001). After performing the propensity score matching, there was no statistically significant difference between the cohorts (Table 1).

**Table 1:** Demographics and clinical characteristics, before and after propensity score matching, in patients with dementia undergoing surgery for displaced femoral neck fractures

	Overall	Before matching		*p* value	After matching		*p* value
	*N* = 9394	pins/screws *N* = 1469	Hemiarthroplasty *N* = 7925		pins/screws *N* = 1466	Hemiarthroplasty *N* = 1466	
Age, mean years (SD)	85 (± 7)	84 (± 9)	85 (± 6)	< 0.001	84 (± 9)	84 (± 7)	0.112
Sex, *n* (%)				< 0.001			0.822
Female	6301 (67.1)	872 (59.4)	5429 (68.5)		872 (59.5)	878 (59.9)	
Male	3093 (32.9)	597 (40.6)	2496 (31.5)		594 (40.5)	588 (40.1)	
Time to surgery, *n* (%)				< 0.001			0.362
Less than 24 h	6074 (64.7)	1025 (69.8)	5049 (63.7)		1022 (69.7)	1001 (68.3)	
More than 24 h	3320 (35.3)	444 (30.2)	2876 (36.3)		444 (30.3)	465 (31.7)	
ASA classification, *n* (%)				< 0.001			1.00
1	92 (1.0)	20 (1.4)	72 (0.9)		20 (1.4)	22 (1.5)	
2	2404 (25.6)	342 (23.3)	2062 (26.0)		342 (23.3)	347 (23.7)	
3	5802 (61.8)	800 (54.5)	5002 (63.1)		800 (54.6)	788 (53.8)	
4	902 (9.6)	266 (18.1)	636 (8.0)		263 (17.9)	277 (18.9)	
5	13 (0.1)	4 (0.3)	9 (0.1)		4 (0.3)	2 (0.1)	
Missing	181 (1.9)	37 (2.5)	144 (1.8)		37 (2.5)	30 (2.0)	
CCI*, *n* (%)				< 0.001			1.00
≤ 4	908 (9.7)	185 (12.6)	723 (9.1)		185 (12.6)	153 (10.4)	
5–6	5788 (61.6)	844 (57.5)	4944 (62.4)		844 (57.6)	860 (58.7)	
≥ 7	2698 (28.7)	440 (30.0)	2258 (28.5)		437 (29.8)	453 (30.9)	
Myocardial infarction, *n* (%)	544 (5.8)	117 (8.0)	427 (5.4)	< 0.001	115 (7.8)	134 (9.1)	0.224
Cerebrovascular disease, *n* (%)	2058 (21.9)	330 (22.5)	1728 (21.8)	0.600	328 (22.4)	313 (21.4)	0.536
Peripheral vascular disease, *n* (%)	309 (3.3)	60 (4.1)	249 (3.1)	0.075	60 (4.1)	60 (4.1)	1.00
Chronic obstructive pulmonary disease, *n* (%)	884 (9.4)	166 (11.3)	718 (9.1)	0.008	165 (11.3)	174 (11.9)	0.638
Congestive heart failure, *n* (%)	1523 (16.2)	284 (19.3)	1239 (15.6)	< 0.001	282 (19.2)	284 (19.4)	0.961
Connective tissue disease, *n* (%)	387 (4.1)	55 (3.7)	332 (4.2)	0.470	55 (3.8)	46 (3.1)	0.421
Diabetes, *n* (%)	1316 (14)	222 (15.1)	1094 (13.8)	0.200	220 (15.0)	205 (14.0)	0.471
Liver disease, *n* (%)	55 (0.6)	16 (1.1)	39 (0.5)	0.010	16 (1.1)	10 (0.7)	0.327
Local tumor, *n* (%)	933 (9.9)	147 (10.0)	786 (9.9)	0.950	147 (10.0)	144 (9.8)	0.902
Metastatic cancer, *n* (%)	152 (1.6)	23 (1.6)	129 (1.6)	0.950	23 (1.6)	14 (1.0)	0.188
Chronic kidney disease, *n* (%)	503 (5.4)	104 (7.1)	399 (5.0)	0.002	102 (7.0)	91 (6.2)	0.444

*CCI was not included when conducting the propensity score matching

*ASA* American Society of Anesthesiologists, *SD* Standard Deviation

There was a higher crude mortality in the pins/screws group compared to the hemiarthroplasty group, both 30 days postoperatively (15.7 vs 12.8%, *p* = 0.003) as well as 1 year postoperatively (43.4 vs 36.1%, *p* < 0.001). After matching the cohorts, the difference in 30-day mortality was no longer significantly different (15.8 vs 14.7%, *p* = 0.434), while 1-year mortality remained higher in the pins/screws group (43.4 vs 37.3%, *p* = 0.001) (Table 2).

**Table 2:** Crude outcomes, before and after propensity score matching, in patients with dementia undergoing surgery for displaced femoral neck fractures

	Before matching		*p* value	After matching		*p* value
	Pins/screws *N* = 1469	Hemiarthroplasty *N* = 7925		Pins/screws *N* = 1466	Hemiarthroplasty *N* = 1466	
LOS			< 0.001			< 0.001
Median (IQR)	6.0 (4.0–9.0)	7.0 (5.0–11.0)		6.0 (4.0–9.0)	7.0 (5.0–10.0)	
Missing	11 (0.7%)	82 (1.0%)		11 (0.8%)	16 (1.1%)	
30-day mortality	231 (15.7%)	1017 (12.8%)	0.003	231 (15.8%)	215 (14.7%)	0.434
1-year mortality	637 (43.4%)	2861 (36.1%)	< 0.001	636 (43.4%)	547 (37.3%)	0.001

*LOS* length of Stay

In the multivariable Poisson regression analysis, prior to propensity score matching, there was a statistically significant association between 30-day postoperative mortality and increased age, male sex, ASA classification above three and congestive heart failure. The incidence of 30-day postoperative mortality after performing hip fracture surgery was not affected by the surgical method used (adj. IRR 0.96, CI 95% 0.83–1.12, *p* = 0.629). This result remained unchanged in the matched cohorts as well (adj. IRR 0.89, CI 95% 0.74–1.09, *p* = 0.286) (Table 3).

**Table 3:** Incidence Rate Ratio (IRR) for 30 day postoperative mortality, before and after propensity score matching, in patients with dementia undergoing surgery for displaced femoral neck fractures

Variable	Before matching		After matching	
	30-day IRR (95% CI)	*p* value	30-day IRR (95% CI)	*p* value
Surgical method				
Pins/screws	ref.		Ref.	
Hemiarthroplasty	0.96 (0.83–1.12)	0.629	0.89 (0.74–1.09)	0.286
Age	1.06 (1.05–1.07)	< 0.001	1.02 (0.95–1.08)	0.604
Sex				
Female	ref.		ref.	
Male	1.71 (1.51–1.92)	< 0.001	2.97 (0.89–9.93)	0.076
Time to surgery				
Less than 24 h	ref.		ref.	
More than 24 h	1.05 (0.93–1.18)	0.447	0.51 (0.15–1.72)	0.279
ASA Classification				
1	ref.		3.7·10^–6^ (3.64·10^–7^–3.86·10^–5^)	< 0.001
2	1.51 (0.57–4.01)	0.413	0.83 (0.54–1.27)	0.387
3	2.10 (0.80–5.52)	0.133	ref.	
4	3.03 (1.14–8.03)	0.026	12.35 (0.39–394.00)	0.155
5	5.28 (1.48–18.80)	0.010	44.98 (0.69–2950.50)	0.075
Myocardial infarction				
No	ref.		ref.	
Yes	1.15 (0.94–1.40)	0.164	2.45 (0.81–7.46)	0.114
Cerebrovascular disease				
No	ref.		ref.	
Yes	1.04 (0.91–1.19)	0.592	0.95 (0.67–1.34)	0.762
Peripheral vascular disease				
No	ref.		ref.	
Yes	1.08 (0.82–1.42)	0.620	1.59 (0.89–2.83)	0.119
Chronic obstructive pulmonary disease				
No	ref.		ref.	
Yes	1.06 (0.88–1.27)	0.576	0.96 (0.54–1.72)	0.900
Congestive heart failure				
No	ref.		ref.	
Yes	1.91 (1.67–2.18)	< 0.001	2.14 (1.41–3.26)	< 0.001
Connective tissue disease				
No	ref.		ref.	
Yes	0.89 (0.66–1.22)	0.492	0.47 (0.21–1.07)	0.071
Diabetes				
No	ref.		ref.	
Yes	1.07 (0.91–1.25)	0.453	1.32 (0.87–2.00)	0.199
Liver disease				
No	ref.		ref.	
Yes	1.49 (0.80–2.76)	0.211	8.39 (0.64–110.63)	0.106
Local tumor				
No	ref.		ref.	
Yes	0.88 (0.73–1.06)	0.182	0.56 (0.30–1.04)	0.066
Metastatic cancer				
No	ref.		ref.	
Yes	1.40 (0.98–2.01)	0.067	0.56 (0.16–1.95)	0.360
Chronic kidney disease				
No	ref.		ref.	
Yes	1.11 (0.91–1.35)	0.325	1.64 (0.90–2.97)	0.106

A Poisson regression model with robust standard errors was used for the unmatched cohorts. A conditional Poisson regression model was used for the matched cohorts adjusting for the same variables. The models were adjusted for age, sex, time to surgery, ASA classification, and comorbidities. Multiple imputation by chained equations was used to account for missing values

*ASA* American Society of Anesthesiologists

**Table 4:** Hazard Ratio (HR) for 1 year postoperative mortality, before and after propensity score matching, in patients with dementia undergoing surgery for displaced femoral neck fractures

Variable	Before matching		After matching	
	1-year HR (95% CI)	*p* value	1-year HR (95% CI)	p value
Surgical method				
Pins/screws	ref.		ref.	
Hemiarthroplasty	0.86 (0.79–0.94)	< 0.001	0.84 (0.74–0.94)	0.002
Age	1.05 (1.05–1.06)	< 0.001	1.05 (1.04–1.06)	< 0.001
Sex				
Female	ref.		ref.	
Male	1.57 (1.46–1.68)	< 0.001	1.46 (1.30–1.66)	< 0.001
Time to surgery				
Less than 24 h	ref.		ref.	
More than 24 h	1.01 (0.94–1.08)	0.807	1.14 (1.00–1.29)	0.043
ASA classification				
1	ref.		0.88 (0.50–1.54)	0.655
2	1.17 (0.77–1.76)	0.462	0.85 (0.73–1.00)	0.051
3	1.51 (1.00–2.28)	0.047	ref.	
4	2.04 (1.34–3.10)	< 0.001	1.41 (1.22–1.63)	< 0.001
5	4.79 (2.27–10.11)	< 0.001	3.36 (1.34–8.39)	0.010
Myocardial infarction				
No	ref.		ref.	
Yes	1.09 (0.96–1.24)	0.194	1.23 (1.01–1.49)	0.036
Cerebrovascular disease				
No	ref.		ref.	
Yes	0.98 (0.91–1.06)	0.647	1.04 (0.90–1.19)	0.608
Peripheral vascular disease				
No	ref.		ref.	
Yes	1.20 (1.02–1.42)	0.031	1.50 (1.16–1.95)	0.002
Chronic obstructive pulmonary disease				
No	ref.		ref.	
Yes	1.11 (0.99–1.23)	0.062	1.08 (0.91–1.29)	0.383
Congestive heart failure				
No	ref.		ref.	
Yes	1.51 (1.39–1.64)	< 0.001	1.46 (1.26–1.68)	< 0.001
Connective tissue disease				
No	ref.		ref.	
Yes	0.86 (0.72–1.02)	0.086	0.87 (0.63–1.20)	0.399
Diabetes				
No	ref.		ref.	
Yes	1.08 (0.99–1.19)	0.095	1.10 (0.93–1.29)	0.273
Liver disease				
No	ref.		ref.	
Yes	1.10 (0.71–1.70)	0.672	0.94 (0.46–1.92)	0.873
Local tumor				
No	ref.		ref.	
Yes	1.07 (0.96–1.18)	0.235	1.15 (0.96–1.39)	0.130
Metastatic cancer				
No	ref.		ref.	
Yes	1.78 (1.44–2.19)	< 0.001	2.24 (1.49–3.36)	< 0.001
Chronic kidney disease				
No	ref.		ref.	
Yes	1.22 (1.07–1.38)	0.003	0.83 (0.67–1.02)	0.075

A Cox proportional hazards model was used for the unmatched cohorts. A shared frailty model was used for the matched cohorts. The models were adjusted for age, sex, time to surgery, ASA classification, and comorbidities. Multiple imputation by chained equations was used to account for missing values

*ASA* American Society of Anesthesiologists

In contrast, when performing the Cox regression analysis on the unmatched cohorts, 1-year postoperative mortality was also associated with chronic kidney disease, peripheral vascular disease and metastatic cancer, in addition to the previously mentioned variables. There was also a 14% reduction in the risk of 1-year postoperative mortality in hemiarthroplasty patients (adj. HR 0.86, CI 95% 0.79–0.94, *p* < 0.001). In the matched cohorts, the risk reduction was 16% for 1-year postoperative mortality among hemiarthroplasty patients (adj. HR 0.84, CI 95% 0.74–0.94, *p* = 0.002) (Table 4).

## Discussion

To the best knowledge of the authors, this is the first study investigating the association between the choice of surgical method, i.e., hemiarthroplasty or pins/screws, and postoperative mortality in patients with dementia and dFNF. The analyses found no difference in the risk of 30-day postoperative mortality between the two surgical interventions; however, hemiarthroplasty was associated with a lower risk of 1-year postoperative mortality. These associations were observed both before and after performing propensity score matching.

The current study specifically focused on patients with dementia since this diagnosis has previously been linked to worse outcomes after hip fracture surgery. A meta-analysis showed that patients with dementia have almost twice the incidence of mortality six months postoperatively compared to patients without dementia [6]. Despite a consensus among orthopedic surgeons that hemiarthroplasty is the preferred surgical method for dFNF, many still choose pins/screws. The authors postulate that this practice is mainly due to the belief of many orthopedic surgeons that arthroplasty will result in a higher perioperative mortality in this older and frailer patient population, due to the increased stress caused by a longer period of time spent under general anesthesia with a more extensive surgical approach and intervention. Using pins/screws is a less invasive method which is thought to minimize tissue damage along with the subsequent inflammatory response. Postoperative systemic inflammation is hypothesized to increase the risk of mortality, particularly from cardiac and respiratory causes [12–15]. This thought process is reflected in the current study since patients who receive pins/screws were generally less fit for surgery measured by their preoperative ASA classification and had more comorbidities.

However, studies have found better functional outcomes in patients who receive arthroplasty compared to internal fixation in terms of mobility, walking aid requirements and postoperative pain [8, 12]. In the study conducted by Rogmark et al., the authors recommend that hemiarthroplasty should be the primary choice of treatment for dFNF in all patients [8]. A prospective study showed that arthroplasty resulted in less pain at four months (34 vs 61%, *p* < 0.001) as well as one year (25 vs 43%, *p* < 0.002) postoperatively when compared to internal fixation in patients who had sustained a dFNF, however, no subgroup analysis was performed for patients suffering from dementia. In the same study, the investigators demonstrated that the arthroplasty group had a significant reduction in walking aid requirements compared to the internal fixation group at four months postoperatively (47 vs 66%, *p* < 0.001) [12]. Furthermore, the arthroplasty group had a better ability to walk up stairs after both 4 months (73 vs 56%, *p* < 0.001) as well as after 2 years (67 vs 54%, *p* = 0.03). The cumulative success rate in the hemiarthroplasty group was considerably higher in the long term, both at one year (93 vs 61%) and two years (92 vs 53%) postoperatively [12]. A primary arthroplasty is both more time-consuming and expensive in the initial phase, but over time, the higher rates of complications associated with internal fixation eliminate any costs saved by this surgical method [16, 17].

The Swedish National Board of Health and Welfare has guidelines recommending the initiation of hip fracture surgery within 24 h of admission [18]. The data from the current study displayed that this was accomplished more often in the pins/screws group compared to the hemiarthroplasty group (69.8 vs 63.7%, *p* < 0.001). This may in part be explained by surgeons preferring to perform hemiarthroplasties during regular office hours due to the greater complexity associated with performing this type of surgery. Another possible reason may be selection bias. There is an increased risk of damage to the arterial blood supply of the femoral head in dFNF. Consequently, if pins/screws are selected as the surgical method, a short time to surgery is essential for reducing the risk of further displacement or injury to the femoral head’s arterial supply, which could cause avascular necrosis. Expediency is not as important when performing hemiarthroplasties since the femoral head is removed and replaced during the course of the operation [19]. These factors should be taken into account when proposing guidelines; the duration of the physiological stress patients’ experience, while awaiting surgery, has been strongly associated with postoperative complications and undesirable outcomes [20].

Before performing any adjustments to the analyses, both 30-day and 1-year mortalities were significantly lower in the hemiarthroplasty cohort compared to the pins/screws cohort. This is in line with the study conducted by Rogmark et al. [8]. When adjusting for relevant covariates, such as comorbidities, there was no statistical difference in 30-day mortality while the risk of 1-year mortality was reduced by 14%. After performing propensity score matching and adjusting for relevant covariates, 30-day mortality remained equivalent in both cohorts while there was a relative risk reduction in 1-year mortality by 16% in patients receiving a hemiarthroplasty.

Approximately 40% of the dFNF patients die within a year after their surgery, which raises the question of how much they actually benefit from hemiarthroplasty. While hemiarthroplasty has been associated with better mobility and less pain, ^12^ more research focusing specifically on patients with dementia is needed. Furthermore, better preoperative tools for selecting the right surgical intervention are needed since many patients with dementia have concomitant cardiovascular diseases, which are highly associated with postoperative complications due to the physiological strain caused by general anesthesia and extensive surgical trauma. These tools would allow for more informed shared decision-making when discussing the choice of surgical method with patients and their relatives.

This study benefitted from a dataset comprised ten years of data from the Swedish national hip fracture database, which is known for having a high case coverage [21]. Patient management is also relatively consistent across treatment centers, due to the universal nature of the Swedish healthcare system. Furthermore, despite the study’s retrospective nature, propensity score matching allowed for the emulation of the benefits associated with prospective randomization [22–24]. Meanwhile, several limitations to the current study are worth mentioning. Due to the retrospective nature of the study, the authors were unable to determine the surgeons’ specific reasoning behind the choice of the selected surgical intervention. Data on cemented versus uncemented hemiarthroplasty were not available in the database for analysis. Postoperative pain, functional outcome, and differences in cost between the cohorts could also not be analyzed. Finally, dementia has been associated with an increased risk of re-interventions due to dislocation after hemiarthroplasty [25]. Re-interventions, however, were not captured in the current database and as such could not be reported. Further research is needed to more clearly illuminate the advantages and disadvantages of hemiarthroplasty versus pins/screws in patients with dementia.

## Conclusion

There was no observable difference in 30-day mortality among patients with dFNF and dementia when comparing pins/screws with hemiarthroplasty. Hemiarthroplasty was, however, associated with a reduced risk of 1-year postoperative mortality.
